# Proximity labeling techniques for protein–protein interaction mapping in plants

**DOI:** 10.1016/j.jbc.2025.110501

**Published:** 2025-07-21

**Authors:** Beyza Özmen, Leonard Blaschek, Michael Ogden, Marcos San Segundo, Staffan Persson, Shuai Zheng

**Affiliations:** 1 Copenhagen Plant Science Center (CPSC), Department of Plant & Environmental Sciences, University of Copenhagen, Frederiksberg C, Denmark; 2 Center for Biopharmaceuticals and Department of Drug Design and Pharmacology, Faculty of Health and Medical Sciences, University of Copenhagen, Copenhagen, Denmark; 3 Joint International Research Laboratory of Metabolic & Developmental Sciences, State Key Laboratory of Hybrid Rice, SJTU-University of Adelaide Joint Centre for Agriculture and Health, School of Life Sciences and Biotechnology, Shanghai Jiao Tong University, Shanghai, China

**Keywords:** protein-protein interactions (PPIs), proximity labeling (PL), plants, TOR complex, cellulose

## Abstract

Protein–protein interactions (PPIs) are fundamental to understanding cellular processes, serving as the cornerstone of biological signaling, structural organization, and metabolic regulation. However, capturing PPIs in living organisms remains a significant challenge, particularly in complex and compartmentalized cellular environments. Research in this area has been greatly accelerated by the invention of proximity labeling (PL) techniques. By employing engineered enzymes capable of tagging proteins and other molecules *in vivo*, PL allows real-time mapping of biomolecular interactions within native environments. In plants, the implementation of PL presents unique challenges but has nonetheless emerged as a powerful tool. Here, we summarize the mechanisms, strengths, and weaknesses of different enzyme-based PL methods. We also highlight key considerations to optimize PL experiments in plants and propose targets for development to further improve their efficiency and flexibility.

Almost all biological processes include complex networks of protein–protein interactions (PPIs), which serve as the molecular machinery driving cellular function. Interactions between proteins vary in both scope and dynamics, ranging from binary interactions to large protein scaffolds, and from transient ‘touch-and-go’ interactions to stable complexes (1). Since functional and physical interactions between proteins often coincide, the functions of undefined and uncharacterized proteins can be approximated by knowing their PPI contexts (2, 3). Thus, it is essential to understand how proteins interact with each other in their natural environments to unravel the complex molecular machinery that governs not only cellular functions but also complex developmental processes (4, 5).

Plants are multicellular organisms that are confronted by several challenges due to their sessile nature. Plant growth and development involve the integration of numerous environmental and endogenous signals (6), and have developed features to respond to them. These adaptations rely on intricate molecular and cellular networks that coordinate biological processes, such as signal transduction, gene regulation, cellular structure, metabolic adjustments, and hormonal responses. While PPIs play critical roles in orchestrating these networks, the unique cellular features of plants, including cell walls, plastids, and complex tissue structures, have proven challenging to characterize PPIs in their native cellular context (4, 7).

Resources like the Search Tool for the Retrieval of Interacting Genes/Proteins (STRING) database enable the exploration of species-specific PPIs (8). By incorporating curated experimental data, alongside co-expression and text-mining analyses, these databases provide powerful predictive tools for investigating potential PPIs. In plants, substantial efforts have been made to elucidate PPIs. For example, the Arabidopsis Interactome Mapping Consortium (2011) used yeast two-hybrid (Y2H) screening to map over 6200 high-confidence PPIs involving ∼2700 proteins (9). These binary interaction maps have identified key connectors and hubs that govern signaling pathways, metabolic processes, and gene regulation. Y2H detects protein interactions by fusing proteins of interest to the split domains of a reporter protein, such as transcription factors or ubiquitin, enabling a simple read-out of PPI (10, 11). By providing a powerful tool to determine PPIs of various species, Y2H is limited to identifying binary interactions that are robust enough to also occur in the non-native context of yeast cells. As such, Y2H generally misses interactions that are conditional on larger protein scaffolds or specific cellular or organellar contexts. Additionally, Y2H is prone to false positive results caused by unspecific aggregation, non-functional protein fusions, and mislocalization of plant proteins in yeast cells. To discover native PPIs more reliably, methods such as Affinity Purification-Mass Spectrometry (AP-MS) (12), co-fractionation MS (13), and cross-linking MS (14) can be used. With these techniques, native protein complexes are co-immunoprecipitated (Co-IP) with the protein of interest (POI) (15), co-fractionated, or stabilized by cross-linkages and subsequently identified by liquid chromatography tandem MS (LC-MS/MS). While these methods are invaluable, they often struggle to capture weak, transient interactions or low-abundance proteins, especially in complex and dynamic cellular environments (4, 14). Moreover, these techniques are susceptible to false-positive PPIs occurring in non-native contexts following cell lysis.

The most consequential recent breakthrough in PPI identification is the development of proximity labeling (PL), which allows researchers to study PPIs in real time under native conditions, with the possibility to detect weak or transient PPIs. This approach leverages enzymes that catalyze reactions to attach labels to proteins in close proximity to the POI, enabling identification using mass spectrometry. PL thereby allows the direct labeling of constitutive and transient PPIs *in vivo*, providing a more comprehensive view of dynamic protein networks than traditional techniques. PL techniques are being increasingly adopted and applied in plant cells.

## Proximity labeling methods for PPIs

**Figure 1:**
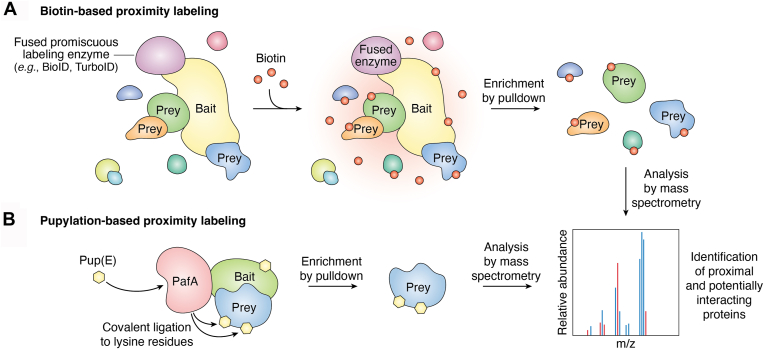
**Overview of proximity labeling (PL) systems.***A*, schematic of biotin-based proximity labeling. A bait protein is genetically fused to a promiscuous labeling enzyme (*e.g.*, BioID or TurboID), which catalyzes the conversion of external biotin into a reactive intermediate. This intermediate diffuses locally and covalently modifies lysine residues on prey proteins, including direct interactors and nearby proteins. Biotinylated proteins can then be enriched by pulldown (*e.g.*, streptavidin-based) and identified by mass spectrometry. *B*, schematic of a pupylation-based proximity labeling system. A bait protein is fused to the bacterial ligase PafA alongside Pup(E), a small peptide substrate. When a prey protein comes into proximity with the PafA-Bait fusion, PafA catalyzes the covalent ligation of Pup(E) to lysine residues on the prey and remains. Pupylated proteins are subsequently enriched and analyzed by mass spectrometry, enabling identification of proximal and potentially interacting proteins.

In general, PL involves a labeling enzyme fused to the POI, often called “bait”. This enzyme catalyzes the covalent attachment of small molecular tags to nearby proteins, known as “preys”. These labeled proteins can be purified and enriched using affinity purification and then analyzed through LC-MS/MS (Fig. 1). In the past few years, a couple of enzymes have been engaged in developing PL, with characteristics inherited from their enzymic mechanisms (Table 1).

**Table 1:** Summary of proximity labeling methods that are described in this review

Approach	Enzyme	Size (kDa)	Substrate	Labeling time	Labeling radius (nm)	Mechanism	Endogenous activity in plant
Ligase-based	BirA∗(BioID)	35	Biotin	24 h	10–20	Diffusion	Yes (low)
	BioID2	27		∼18 h			Yes (low)
	Split-BioID	35		∼18 h			Yes (lower than BirA∗)
	TurboID	35		≥10 min	5–10	Diffusion	Yes (high)
	miniTurbo	28					Yes (lower than TurboID)
	Split-TurboID	35					Yes (lower than full TurboID)
Peroxidase-based	HRP	44	Biotin-phenol + H_2_O_2_	10 min to 2 h	<200–300	Diffusion	Yes (high)
	APEX	28		1 min	20	Diffusion	Yes (high)
	APEX2						Yes (high)
	Split-APEX						Yes (lower than full APEX)
Pupylation-based (PUP-IT)	PafA	54	Pup and derivative peptides	6–24 h	<10 nm	Ligation	No

## Biotin ligase-based PL techniques

Biotin ligase-based labeling is one of the most widely used techniques to study direct and indirect interactions between proteins *in vivo* due to its simplicity, versatility, and effectiveness (Fig. 1*A*). Numerous studies have successfully applied the biotin-ligase PL method in different organisms to study PPIs, including yeast (16), *Drosophila* (17), zebrafish (18), mice (19), and plants (20, 21, 22). In 2004, Choi-Rhee *et al.* reported a mutant biotin protein ligase, BirA∗ (R118G), from *Escherichia coli*, which exhibited a dramatically increased dissociation constant for activated biotin-5′-AMP (23). As a result, BirA∗ R118G freely released biotin-5′-AMP into the surrounding medium, enabling unspecific, diffusion-constrained covalent labeling of nearby proteins. The first biotin ligase-based PL technique, BioID, was based on fusing this enzyme to bait (5). Upon addition of exogenous biotin, the biotin ligase generates activated biotin-5′-AMP, which labels lysine residues in prey within an effective diffusion range of ∼10 nm around the bait (24, 25). The biotinylated proteins can be captured and enriched by streptavidin beads, purified, and analyzed by mass spectrometry. However, the R118G mutation also reduces the affinity for biotin, which may lead to decreases in biotinylation in the absence of sufficient biotin (26); therefore, excess biotin is required to enable continual prey biotinylation. Additionally, the size of BirA (35 kDa) can interfere with the proper folding or function of the bait protein. To mitigate this issue, Kim *et al.* (2016) (27) developed BioID2 (27 kDa) from the smaller biotin ligase of the thermophile *Aquifex aeolicus*. Labeling promiscuity was achieved through an arginine to glycine mutation (R40G), analogous to the R118G mutation in BirA∗. The reduction in size aimed to decrease interference with bait folding and function, improving proximity labeling accuracy. Additionally, BioID2 requires less biotin supplementation and shorter labeling time while maintaining efficient prey biotinylation (28). Still, the labeling duration (∼16 h) can hinder its ability to capture dynamic interactions and may increase the likelihood of non-specific labeling over time. To overcome these limitations, Branon *et al.* (2018) (29) introduced TurboID (35 kDa) and miniTurboID (28 kDa), two efficient alternatives to the BioID system that reduced the labeling time from several hours to just 10 min. Using an error-prone PCR mutagenesis screen of BirA-R118S, which is approximately twofold more active than R118G, they identified several mutants with significantly faster biotinylation kinetics through yeast display-based directed evolution. Consequently, TurboID significantly accelerates the biotinylation process while maintaining the labeling radius of BioID. The smaller miniTurboID retains high biotinylation activity and is particularly useful when fusing the ligase to small proteins or targeting it to compact cellular compartments; however, it is less stable than TurboID due to the lack of its N-terminal domain. TurboID and miniTurboID were validated in a variety of biological systems, including mammalian cells, *Drosophila melanogaster,* and *Caenorhabditis elegans* (29).

Continuous development and quick adoption have made biotin-ligase-based approaches the primary PL technique in plant science. Khan *et al.* (2018) (30) first demonstrated the successful application of BioID in leaf tissues of the model plant *Arabidopsis thaliana*, showcasing its potential as an addition to the proteomic toolbox for plant research. Subsequently, Mair *et al.* (20) showed that both TurboID and miniTurboID provided higher sensitivity than BioID in infiltrated *Nicotiana benthamiana* (a model tobacco plant) leaves and transformed *A. thaliana* seedlings. Mair *et al.* (20) also compared the required biotin concentrations, labeling times, and temperatures between TurboID and miniTurboID, where TurboID showed higher efficiency, while miniTurboID had an advantage in terms of specificity. Since then, variants of TurboID have been used to profile various plant interactomes, such as Arabidopsis meiotic chromosome axes (31), BRASSINOSTEROID-INSENSITIVE 2 (BIN2) kinase (32), and the target of rapamycin (TOR) complex (33). In the BIN2 study, TurboID was fused to the kinase to identify *in vivo* interaction partners, revealing over 480 proximal proteins and demonstrating strong enrichment for BIN2-dependent phosphoproteins. Furthermore, TurboID was fused to SnRK1/TOR core complex subunits and used to uncover nitrogen-specific interactors, revealing novel connections between SnRK1 and ER-localized stress responses in the TOR signaling. However, one of the key limitations of these systems is their susceptibility to high background labeling *in planta* due to endogenous biotinylation. Plants naturally produce and utilize biotin, leading to constitutive biotinylation of native proteins independent of the introduced ligase. This caveat notwithstanding, biotin ligase-based PL techniques will likely continue to improve and remain a cornerstone for research on plant PPIs.

## Peroxidase-based PL techniques

Peroxidase-based PL techniques have been used as significant labeling tools for mapping PPIs, mainly in mammalian tissues and cell cultures. These techniques, named APEX (34) and APEX2 (35), are based on engineered plant ascorbate peroxidases. Upon exogenous addition of phenol-biotin and H_2_O_2_, the peroxidase fused to the POI reduces H_2_O_2_ to oxidize phenol-biotin molecules into highly reactive radicals. The phenol-biotin radicals then diffuse short distances before reacting with the phenolic side chain of tyrosine residues (and to some extent other electron-rich amino acids) in nearby proteins, resulting in an effective labeling radius of approximately 20 nm. However, the adoption of APEX2 in plant science has been hamstrung by three significant drawbacks: the toxicity of exogenous H_2_O_2_, the limited membrane-permeability of phenol-biotin, and, crucially, the high background labeling by endogenous peroxidases with activity towards phenolic substrates, which are ubiquitous in plants (36). Although the capacity of APEX2 to polymerize electron dense substrates has recently been exploited to localize APEX2 fusion proteins in electron micrographs (37), the successful use of peroxidase-based PL in plants has yet to be demonstrated. In addition to APEX2, horseradish peroxidase (HRP) has emerged as a promising peroxidase-based PL tool for labeling proteins at cell-cell contact sites. Recently, it was demonstrated that membrane-anchored HRP can use endogenously generated extracellular H_2_O_2_ to selectively label neighboring interacting cells under physiological conditions, enabling non-invasive mapping of cell-cell networks without exogenous H_2_O_2_ treatment (38).

## Pup ligase-based PL techniques

In 2018, Liu *et al.* introduced a new proximity labeling system in mammalian cells called pupylation-based interaction tagging (PUP-IT) (39). This system is based on pupylation, analogous but unrelated to ubiquitination in eukaryotes, and involves the C-terminal deamination and subsequent covalent attachment of a small 7-kDa prokaryotic ubiquitin-like protein (Pup) to lysine residues on target proteins by the ligase PafA (Proteasome Accessory Factor A) (Fig. 1*B*). PUP-IT uses this bacterial pupylation system to tag proteins that are in close proximity to the bait protein, detecting PPIs in living cells. The PUP-IT technique involves expressing a POI fused to PafA alongside deaminated Pup (PupE) (40). PafA uses ATP to activate PupE, forming a reactive γ-glutamyl phosphate intermediate at its C-terminus (41). Activated PupE remains tightly bound to PafA as a non-diffusive intermediate until conjugation occurs, minimizing non-specific labeling and ensuring highly localized tagging of proteins in direct proximity of the bait. This intermediate is ligated to lysine residues on preys, resulting in a stable isopeptide bond between PupE and prey. Subsequently, PupE-tagged proteins can be enriched and identified by mass spectrometry. Despite its recent development, improvements and modifications to PUP-IT have already emerged. In PUP-IT2, PupE is fused to the POI instead of PafA, allowing free PafA to catalyze the covalent attachment of PupE between the POI and its interacting proteins. When fused to the POI, PupE (7 kDa) is less likely to impair native protein folding than PafA (55 kDa), making this variant a promising alternative for structurally sensitive proteins (42). Since its relatively recent inception, PUP-IT has emerged as the primary alternative to biotin ligase-based approaches for PL in plant science. Because pupylation, unlike biotinylation and peroxidase-based methods, is absent from plant metabolism, PUP-IT exhibits lower background labeling compared to other PL methods. The PUP-IT approach has so far been applied in several plant systems to uncover PPIs in biological relevant contexts. In the Arabidopsis protoplast transient expression system, PUP-IT was used to confirm a substrate of the TOR kinase, highlighting its potential for mapping signaling pathways (43). More recently, PUP-IT was successfully adapted to map PPIs of the plasma membrane-localized receptor kinase FERONIA (44), the regulatory networks involved in cellulose biosynthesis (45), and protein interactomes of the TOR complex (46). In three cases, PUP-IT successfully recovered an abundance of both known and novel interactors, validating its utility for plant signaling and developmental studies. Additionally, Zheng *et al.* (45) compared the capacity of PUP-IT and TurboID to recover known interactors of the TPLATE complex that mediates membrane bending during endocytosis. PUP-IT offered higher specificity, whereas TurboID captured a broader range of proximate proteins, reflecting the mechanistic differences between the two systems.

In its current form, PUP-IT requires longer labeling times (>4 h) than TurboID. Nonetheless, the absence of exogenously added substrates and the lack of label diffusion make PUP-IT a promising technique with unique advantages to other PL techniques. To enhance purification and detection of labeled proteins, modified Pup(E) substrates, such as FLAG-Pup(E), StrepII-FLAG-Pup(E) (45), biotinylated Pup(E), and a shortened DE28 variant (39), have been employed, enabling efficient enrichment using streptavidin or antibody-based pulldown under denaturing or native conditions. These tags, often co-expressed with PafA from the same vector, streamline the experimental setup and enhance the flexibility and applicability of PUP-IT in diverse systems. The mechanistic and structural insights of PafA enable the rational engineering variants with enhanced activity and conditional versions, broadening the toolbox for PL (40). Since the PUP-IT system is entirely genetically encoded, it can be seamlessly integrated with other genetic tools. These might open promising pathways for spatio-temporal control of interactome mapping, especially *via* integration with chemically inducible dimerization systems, optogenetics, and CRISPR-base targeting strategies.

## Experimental considerations for PL in plants

**Figure 2:**
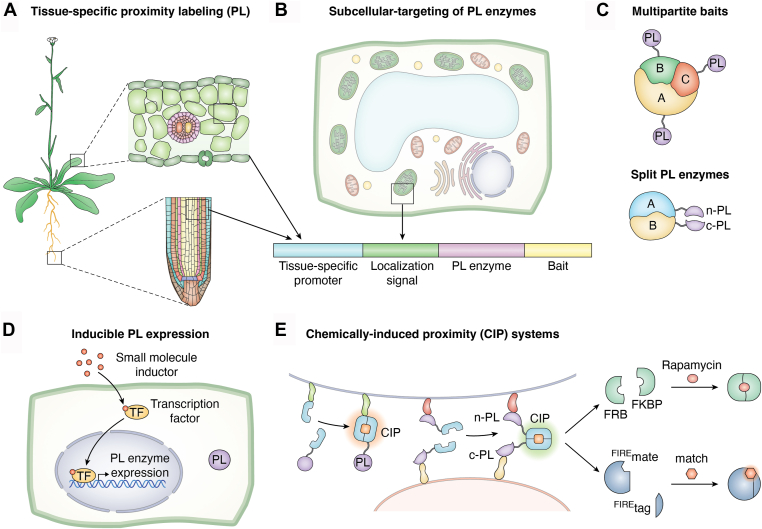
**Experimental considerations to optimize proximity labeling (PL) in plants.***A*, tissue-specific PL expression. Schematic representation of *Arabidopsis thaliana* model plant with zoomed-in views of leaf and root cells where PL constructs can be expressed under tissue-specific promoters. *B*, subcellular targeting of PL enzymes. PL enzymes can be directed to specific organelles or membrane compartments by fusing them to well-characterized localization signals. A schematic plant cell illustrates representative compartments such as the chloroplast, mitochondria, nucleus, and cytosol. The example of construct design includes a promoter, localization signal, PL enzyme, and bait protein to enable spatially restricted protein labeling. *C*, multipartite baits and split PL strategies. PL enzymes are individually fused to different subunits of a known protein complex (*A*, *B*, and *C*) to capture subunit-specific interactomes, which enables mapping of differential proximity environments within the same complex (*upper panel*). The *lower panel* shows the split-PL enzyme strategy, where two interacting proteins (*A* and *B*) are fused to N-terminal and C-terminal complementary fragments of a split PL enzyme n-PL and c-PL). Upon complex formation, the proximity of the two fusion partners (*A* and *B*) allows reconstitution of enzymatic activity, leading to labeling of nearby proteins. *D*, inducible PL expression for precise temporal and spatial control over PL activity. The schematic view of transcriptional control of PL expression that relies on small molecule-inducible transcription factors (*e.g.*, DEX-responsive TF), enabling conditional PL activation. *E*, chemically induced proximity (CIP) systems. PL activity can be triggered *via* chemically induced dimerization of fusion proteins by bringing them into proximity for labeling. It enables controlled recruitment of a full-length PL enzyme to a protein of interest or reconstitution of a split PL enzyme. CIP concept could be different; the *upper panel* depicts the FKBP/FRB system activated by rapamycin. The *lower panel* illustrates the concept of the recently developed CATCHFIRE system, which relies on the ^FIRE^tag and ^FIRE^mate that can interact in the presence of the fluorogenic inducer, match, to assemble.

PL is a versatile technique for studying PPIs and mapping protein networks. To obtain reliable and high-quality results, some critical parameters should be taken into consideration when designing PL experiments. To ensure capturing native interactomes, functionality of the bait protein fused to a PL enzyme should be confirmed by complementation of a corresponding loss-of-function mutant, and known interactors should be identified as positive controls following LC-MS/MS. Beyond these fundamental considerations, PL approaches can be adapted to restrict labeling spatially and temporally, providing deeper insights into dynamic protein networks within specific cellular contexts (Fig. 2).

## Expression—broad or cell type-specific?

For widely expressed POIs, using the native promoter is often the obvious choice (31, 44). For less abundant proteins, widely expressed promoters like that of Ubiquitin10 (45, 46) or the cauliflower mosaic virus 35S (32) can be used to improve labeling efficiency. Tissue-specific promoters enable PL to be performed in a subset of cells within complex tissues, ensuring high-resolution protein interaction mapping *in vivo* (Fig. 2*A*). For example, Mair *et al.* (20) demonstrated the successful application of TurboID and miniTurboID by picking a rare protein and cell type in plants. By fusing TurboID to the guard-cell-specific transcription factor FAMA, expressed under its native promoter, they identified previously unknown FAMA interactors despite FAMA’s low abundance. This approach highlights the potential of TurboID for cell-type-specific proteomics in plants. Whether the labeling efficiency of PUP-IT is sufficient for such approaches is yet to be shown. Nevertheless, its enzymatic mechanism and potential for higher specificity make PUP-IT a highly promising approach for cell-type-specific PPI mapping, especially when combined with optimized expression strategies and controlled labeling conditions.

## Subcellular localization

Besides specific cell types, PL can be focused on specific subcellular compartments such as membrane fractions, cytosol, or organelles. The simplest approach for such targeting is unrestricted PL (without specific subcellular targeting), followed by protein fractionation to enrich interactors localized in the targeted compartment (*e.g.* microsomes, nuclei, *etc.*) (44). Alternatively, PL enzymes can be fused to specific localization signals, target peptides, or membrane anchors, limiting the PL reactions to the respective compartment (Fig. 2*B*). For example, targeting PL enzymes to the nucleus (20, 47), mitochondria (48), chloroplasts (49), or endoplasmic reticulum (50) allows mapping of PPIs within these compartments while minimizing background from cytosolic proteins. Here, care must be taken to ensure the availability of the biotin or PupE substrates in the respective compartment. Biotin diffuses into organelles relatively freely, and—at least in surface tissues—efficiently labels proteins within compartments such as plastids (51, 52) and nuclei (20). For efficient pupylation in organelles, PupE also needs to be targeted to the compartment of interest with the respective localization signal. Organelle-specific PUP-IT has yet to be demonstrated but could potentially offer better substrate availability in deeper tissues, due to its fully endogenous mechanism.

## Multipartite baits and split PL enzymes

What if we want to identify PPIs of a protein complex, *i.e.* multipartite interactions, instead of a single protein of interest? One approach is to fuse PL enzymes to individual subunits of a known protein complex and compare the resulting interactors (Fig. 2*C*). For example, the interactome of the TOR complex (TORC) was recently characterized by integrating data from experiments targeting TORC subunits LST8-1 and RAPTOR1, as well as TORC suppressor FKBP (46). Alternatively, split enzyme approaches make it possible to restrict PL reactions to the site of a specific, previously determined protein complex, by fusing two complementary parts of the PL enzyme to two complex-forming proteins (Fig. 2*C*). Only when the proteins come together to form a complex will the PL enzyme reconstitute into its functional form and label interactors. In 2017, a BirA∗ was split at three distinct positions within flexible loop regions of the catalytic domain to avoid disrupting secondary structure elements. The split at E140/Q141 was used to specifically label targets of a phosphatase complex following dimerization since it produced a detectable biotinylation signal (53). Independently, Schopp *et al.* (54) identified an alternative split site at E256/G257, just before the final helix of the catalytic core, which exhibited low background activity and robust reconstitution upon rapamycin-induced dimerization. This approach offered advantages over BioID by capturing transient or weak interactions of the complex, while excluding interactions of the individual proteins during maturation or turnover. Nonetheless, the relatively slow biotinylation kinetics of BioID (∼16 h) again limit its ability to detect highly dynamic interactions and increase the likelihood of non-specific labeling over time. This drawback was addressed with the introduction of split-TurboID, reducing labeling times from 16 to 4 h (55). An efficient split-site for the PUP-IT PafA enzyme is yet to be identified. However, the flexible linker between the large N-terminal domain and the small 70 aa C-terminal domain of PafA (T412–R418) appears to be a promising candidate site for an efficient split, since it would disrupt the ATP binding site (40), while likely maintaining efficient folding of the two domains.

## Substrate supply and labeling time

Controlling the duration of labeling is a key parameter for optimization. Shorter labeling times capture interactions of abundant proteins and minimize background labeling. Conversely, low abundant proteins or transient interactions may require extended labeling periods (20). In biotin-ligase-based systems, the exogenous biotin supply makes it possible to manipulate the process to a desired scale. However, this is contingent on efficient substrate uptake by the organism. In some cases, poor permeability or compartmental restrictions may limit substrate availability, reducing labeling efficiency. Conversely, plants naturally synthesize and accumulate biotin, which can lead to background labeling even in the absence of supplementation. Particularly in highly active systems such as TurboID, there is an increased risk of nonspecific labeling or spurious protein identification, complicating data interpretation. Inducible promoter systems allow for controlled gene expression and might partially mitigate this by restricting PL enzyme expression to defined time periods. These systems often rely on small molecules such as estradiol, dexamethasone, or tetracycline, which bind to a regulatory protein (transcription factor, TF), triggering transcriptional activation (56) (Fig. 2*D*). In the context of PL, inducible systems can be used to control the expression of the fused PL enzyme (55), or, in the case of the fully proteinaceous PUP-IT system, the expression of the PupE substrate. While useful for reducing background and enabling temporal resolution, these systems may exhibit limitations, including leaky background expression, tissue-specific variability in induction efficiency, or delayed transcriptional activation relative to the timescale of protein interactions. Thus, careful optimization of substrate delivery, labeling duration, and induction kinetics is essential to maximize specificity and sensitivity in PL-based interactome profiling.

## Spatiotemporal control through chemically induced proximity

Conventional PL techniques rely on untargeted labeling of PPIs. Chemically induced proximity (CIP) labeling is emerging as a powerful complementary approach, offering precise spatio-temporal control for PL events. By using small molecule-induced dimerization, CIP could allow controlled recruitment of a PL enzyme to a POI or bring a split PL enzyme together (Fig. 2*E*) as discussed above, thereby enabling precise and tunable control over labeling.

One of the earliest and most widely used CIP systems involves the macrolide compound rapamycin, which induces strong dimerization between FK506-BINDING PROTEIN (FKBP) and the FKBP-rapamycin binding (FRB) domain of TOR. By fusing one POI to FKBP and another to FRB, dimerization is induced upon rapamycin application (Fig. 2*E*). Lee *et al.* (57) demonstrated the potential of rapamycin-directed CIP to complement PL by fusing BirA∗ to FRB and using rapamycin to induce dimerization in human embryonic kidney 293T (HEK293T) cells. This approach enabled biotinylation of rapamycin-induced FRB-interacting proteins and their identification *via* mass spectrometry following streptavidin-based enrichment.

Rapamycin-induced CIP has also been widely used as a validation tool in the development of split PL systems, including split-HRP (58), split-APEX2 (59), split-BioID (54, 60), and split-TurboID (55). In these systems, split PL enzyme fragments are fused to FKBP and FRB, and rapamycin is used to induce their dimerization, followed by assessment of enzymatic activity. Cho *et al.* (55) further advanced rapamycin-directed CIP with PL by fusing split-TurboID fragments to FKBP and FRB along with subcellular localization sequences. Although not as active as full-length TurboID, rapamycin-directed split-TurboID enabled spatial and temporal control of PL in HEK293T cells within 30 min following rapamycin treatment.

In plants, rapamycin-directed CIP has been successfully employed in *A. thaliana* and *N. benthamiana*, which gain sensitivity to rapamycin when expressing heterologous human- or yeast-derived FKBP (61, 62, 63, 64). Winkler *et al.* (64) examined transient expression of FKBP- and FRB-fused proteins and fluorophores in *N. benthamiana* leaves. Not only did rapamycin treatment (1 μM) trigger protein dimerization within minutes, but FKBP-fused proteins could also be targeted to distinct subcellular compartments through the fusion of anchor domains to FRB. Moreover, in exploring the interactome of the TOR complex (TORC) in Arabidopsis, rapamycin achieved targeting the yeast FKBP fused proximity labelling enzymes to TOR kinase (46).

A unique application of CIP with PL emerged in the form of a modified PUP-IT system, called POST-IT (Pup-On-target for Small molecule Target Identification Technology) (65). Designed for drug target screening, POST-IT takes advantage of the affinity of bioactive small molecules for their protein targets. In this system, PafA is fused to the self-labelling enzyme HaloTag, while the small molecule of interest is derivatized with a HaloTag chloroalkane ligand. This modification enables the small molecule to simultaneously bind its protein target and HaloTag-PafA, effectively recruiting PafA to the target protein to facilitate proximity labeling. The authors demonstrated POST-IT in both HEK293T cells and zebrafish embryos, establishing that HaloTag ligand-derivatized small molecules can successfully induce CIP to direct PL in live cells, expanding the CIP-PL toolbox beyond rapamycin-directed systems.

A recent advance in CIP systems was developed in 2023, coined CATCHFIRE (chemically assisted tethering of chimera by fluorogenic-induced recognition) (66). Designed and tested in human cell lines, CATCHFIRE incorporates two small proteins called ^FIRE^tag (11 amino acids) and ^FIRE^mate (114 amino acids), derived from bisected pFAST (promiscuous Fluorescence-Activating and absorption-Shifting Tag) (67), a fluorescent chemogenetic reporter. Two proteins of interest are fused with ^FIRE^tag and ^FIRE^mate, and dimerization is induced by the addition of a small-molecule fluorogen, termed “match,” which gains fluorescence upon complex formation (Fig. 2*E*). This CIP system has the added benefit of tracking PPIs *in vivo* using fluorescence microscopy. Upon the addition of sub-micromolar concentrations of match, CIP occurs within minutes. A key feature of CATCHFIRE is its reversibility; removing the match compound by washout dissociates the interacting proteins, providing a powerful tool for studying transient and dynamic interactions. Although not yet reported as implemented in plants, this CIP system offers a unique method for probing and modulating PPIs.

Using the approaches outlined earlier, CIP could be used to achieve precise spatio-temporal control over PL, enabling targeted labeling of specific POIs or induced protein complexes. Additionally, CIP strategies could direct PL enzymes to specific subcellular compartments or regulate labeling duration through inducible enzyme reconstitution. As these systems continue to evolve and are adapted for plant research, they hold great promises for expanding our ability to study and manipulate PPIs with unprecedented precision.

## Data analysis

The combined details of proteomic analysis approaches for plant samples go beyond the scope of this text. However, there are certain specific adjustments of standard approaches that can improve the quality of the PL data. Because we expect considerable amounts of missing data in PL samples from bait-specific preys—*i.e.* proteins interacting with one bait but being entirely missing from another bait – data-dependent acquisition is usually a reasonable strategy that yields easily interpretable data. Data-independent acquisition increases sensitivity and resolution but requires more careful analysis. For peptide searches, biotinylation (+226.078 Da) (68) or pupylation (+243.086 Da, assuming trypsin digestion) (69) should be added as variable modifications. This allows identification of biotinylated or pupylated sites, which further verifies successful labeling and, in very abundant proteins, could even yield information about which part of a prey protein is proximate to the fused PL enzyme. Additionally, biotinylation and pupylation of lysine residues inhibit their tryptic cleavage, leading to large numbers of missed cleavages in PL samples. To avoid missing heavily labeled proteins, the search algorithm should allow for at least two missed cleavages. All of the above is easily adjustable using tools such as the powerful open source project FragPipe (70, 71). For the subsequent differential enrichment analysis, a robust algorithm for the handling of randomly and non-randomly missing data is required. Generally, mixed imputation approaches are recommended (72), but missing-data-tolerant approaches like MSqRob-hurdle might also be worth considering (73). Mixed imputation approaches are widely used and allow sufficient true positive rates even with relatively limited numbers of replicates (45, 46). Missing-data-tolerant approaches, on the other hand, do not require us to make assumptions about the causes behind missing data, thereby minimizing false discovery rates (73). The final, and crucial, requirement for meaningful differential enrichment analysis is an appropriate control bait. Using a freely diffusing protein such as GFP, targeted to the same subcellular localization as the POI, is the simplest solution. However, when investigating the specificity of certain interactions, it might be worth using several related and/or co-localized proteins as controls that can provide a more refined assessment of specificity. For example, when studying interactions within a protein complex, a structurally related but functionally distinct paralog could serve as a valuable comparison. Likewise, for proteins involved in membrane trafficking, unrelated membrane-associated protein may help differentiate between general vesicle interactions and specific binding partners. Ultimately, the choice of control is critical and should be guided by the biological context and question of the experiment to ensure robust and biologically meaningful results.

## Conclusion and perspective

Proximity labeling is complementary to more traditional methods such as Y2H or Co-IP, each offering their own strengths and weaknesses for studying PPIs. PL techniques have been widely applied across various research areas and biological systems to unravel complex cellular processes. PL methods are powerful, accessible, simple, capture dynamic interactions, detect weak or transient interactions, and help to map protein networks. This rapidly advancing field provides researchers with valuable new insights into the cellular processes and their regulation from a proteome-wide perspective. However, each PL method has distinctive drawbacks. BioID and TurboID are diffusion-based, limited by their reliance on exogenous biotin, which may not be uniformly accessible across all experimental systems, and suffer from background labeling noise. PUP-IT, although advantageous in reducing experimental artifacts, exhibits slower labeling kinetics. These limitations underscore the need for continued refinement and innovation in PL technologies. Recent advancements, such as POST-IT, demonstrate how strategic modifications can enhance specificity and improve efficiency in biological applications (65). However, a shortening of labeling times, akin to those achieved through the development of TurboID, is yet to be shown for pupylation-based systems. These current and future developments, together with breakthroughs in photocatalyst-based PL covered elsewhere (74), mean that the molecular toolbox for PL is sure to grow in the coming years, offering ever more sophisticated approaches to decipher the dynamics of PPIs in living plants. As labeling enzymes become faster, more selective, and better adapted for plant use, PL will be increasingly applied to dissect complex signaling cascades, investigate cell- or stimulus-specific responses, and resolve interaction networks with spatial and temporal resolution. Integration of PL with inducible systems, conditional labeling, or emerging -omics platforms will further enhance its capacity to reveal how protein networks are dynamically rewired during development and environmental adaptation.

## Conflict of interest

The authors declare that they do not have any conflicts of interest with the content of this article.

## References

[1] De Las Rivas J., Fontanillo C. (2010). Protein–protein interactions essentials: key concepts to building and analyzing interactome networks. PLoS Comput. Biol..

[2] von Mering C., Krause R., Snel B., Cornell M., Oliver S.G., Fields S. (2002). Comparative assessment of large-scale data sets of protein–protein interactions. Nature.

[3] Pedamallu C.S., Posfai J. (2010). Open source tool for prediction of genome wide protein-protein interaction network based on ortholog information. Source Code Biol. Med..

[4] Xu S.-L., Shrestha R., Karunadasa S.S., Xie P.-Q. (2023). Proximity labeling in plants. Annu. Rev. Plant Biol..

[5] Roux K.J., Kim D.I., Raida M., Burke B. (2012). A promiscuous biotin ligase fusion protein identifies proximal and interacting proteins in mammalian cells. J. Cell Biol..

[6] Leisner C.P., Potnis N., Sanz-Saez A. (2023). Crosstalk and trade-offs: plant responses to climate change-associated abiotic and biotic stresses. Plant Cell Environ..

[7] Rhee S.Y., Mutwil M. (2014). Towards revealing the functions of all genes in plants. Trends Plant Sci..

[8] Szklarczyk D., Kirsch R., Koutrouli M., Nastou K., Mehryary F., Hachilif R. (2023). The STRING database in 2023: protein–protein association networks and functional enrichment analyses for any sequenced genome of interest. Nucleic Acids Res..

[9] Dreze M., Carvunis A.-R., Charloteaux B., Galli M., Pevzner S.J., Arabidopsis Interactome Mapping Consortium (2011). Evidence for network evolution in an *Arabidopsis* interactome map. Science.

[10] Fields S., Song O. (1989). A novel genetic system to detect protein–protein interactions. Nature.

[11] Ito T., Ota K., Kubota H., Yamaguchi Y., Chiba T., Sakuraba K. (2002). Roles for the two-hybrid system in exploration of the yeast protein interactome. Mol. Cell. Proteomics.

[12] Liu X., Salokas K., Weldatsadik R.G., Gawriyski L., Varjosalo M. (2020). Combined proximity labeling and affinity purification−mass spectrometry workflow for mapping and visualizing protein interaction networks. Nat. Protoc..

[13] McWhite C.D., Papoulas O., Drew K., Cox R.M., June V., Dong O.X. (2020). A pan-plant protein complex map reveals deep conservation and novel assemblies. Cell.

[14] Young M.M., Tang N., Hempel J.C., Oshiro C.M., Taylor E.W., Kuntz I.D. (2000). High throughput protein fold identification by using experimental constraints derived from intramolecular cross-links and mass spectrometry. Proc. Natl. Acad. Sci. U. S. A..

[15] Lin J.-S., Lai E.-M., Journet L., Cascales E. (2017). Bacterial Protein Secretion Systems: Methods and Protocols.

[16] Larochelle M., Bergeron D., Arcand B., Bachand F. (2019). Proximity-dependent biotinylation mediated by TurboID to identify protein–protein interaction networks in yeast. J. Cell Sci.

[17] Zhang B., Zhang Y., Liu J.-L. (2021). Highly effective proximate labeling in Drosophila. G3 GenesGenomesGenetics.

[18] Rosenthal S.M., Misra T., Abdouni H., Branon T.C., Ting A.Y., Scott I.C. (2021). A toolbox for efficient proximity-dependent biotinylation in zebrafish embryos. Mol. Cell. Proteomics.

[19] Takano T., Wallace J.T., Baldwin K.T., Purkey A.M., Uezu A., Courtland J.L. (2020). Chemico-genetic discovery of astrocytic control of inhibition in vivo. Nature.

[20] Mair A., Xu S.-L., Branon T.C., Ting A.Y., Bergmann D.C. (2019). Proximity labeling of protein complexes and cell-type-specific organellar proteomes in Arabidopsis enabled by TurboID. eLife.

[21] Arora D., Abel N.B., Liu C., Van Damme P., Yperman K., Eeckhout D. (2020). Establishment of proximity-dependent biotinylation approaches in different plant model systems[OPEN]. Plant Cell.

[22] Li X., Wei Y., Fei Q., Fu G., Gan Y., Shi C. (2023). TurboID-mediated proximity labeling for screening interacting proteins of FIP37 in Arabidopsis. Plant Direct..

[23] Choi-Rhee E., Schulman H., Cronan J.E. (2004). Promiscuous protein biotinylation by Escherichia coli biotin protein ligase. Protein Sci..

[24] Santos-Barriopedro I., van Mierlo G., Vermeulen M. (2021). Off-the-shelf proximity biotinylation for interaction proteomics. Nat. Commun..

[25] Samavarchi-Tehrani P., Samson R., Gingras A.-C. (2020). Proximity dependent biotinylation: key enzymes and adaptation to proteomics approaches∗. Mol. Cell. Proteomics.

[26] Li P., Li J., Wang L., Di L.-J. (2017). Proximity labeling of interacting proteins: application of BioID as a discovery tool. PROTEOMICS.

[27] Kim D.I., Jensen S.C., Noble K.A., Kc B., Roux K.H., Motamedchaboki K. (2016). An improved smaller biotin ligase for BioID proximity labeling. Mol. Biol. Cell.

[28] Kim D.I., Roux K.J. (2016). Filling the void: proximity-based labeling of proteins in living cells. Trends Cell Biol..

[29] Branon T.C., Bosch J.A., Sanchez A.D., Udeshi N.D., Svinkina T., Carr S.A. (2018). Efficient proximity labeling in living cells and organisms with TurboID. Nat. Biotechnol..

[30] Khan M., Youn J.-Y., Gingras A.-C., Subramaniam R., Desveaux D. (2018). In planta proximity dependent biotin identification (BioID). Sci. Rep..

[31] Feng C., Roitinger E., Hudecz O., Cuacos M., Lorenz J., Schubert V. (2023). TurboID-based proteomic profiling of meiotic chromosome axes in Arabidopsis thaliana. Nat. Plants.

[32] Kim T.-W., Park C.H., Hsu C.-C., Kim Y.-W., Ko Y.-W., Zhang Z. (2023). Mapping the signaling network of BIN2 kinase using TurboID-mediated biotin labeling and phosphoproteomics. Plant Cell.

[33] Persyn F., Smagghe W., Eeckhout D., Mertens T., Smorscek T., Winne N.D. (2024). A nitrogen-specific interactome analysis sheds light on the role of the SnRK1 and TOR kinases in plant nitrogen signaling. Mol. Cell. Proteomics.

[34] Rhee H.-W., Zou P., Udeshi N.D., Martell J.D., Mootha V.K., Carr S.A. (2013). Proteomic mapping of mitochondria in living cells via spatially-restricted enzymatic tagging. Science.

[35] Lam S.S., Martell J.D., Kamer K.J., Deerinck T.J., Ellisman M.H., Mootha V.K. (2015). Directed evolution of APEX2 for electron microscopy and proximity labeling. Nat. Methods.

[36] Blaschek L., Pesquet E. (2021). Phenoloxidases in plants—how structural diversity enables functional specificity. Front. Plant Sci..

[37] Lampugnani E.R., Persson S., van de Meene A.M.L. (2024). Colocalising proteins and polysaccharides in plants for cell wall and trafficking studies. Front. Plant Sci..

[38] Cho Y., Jeong I., Kim K., Rhee H.-W. (2025). Painting cell–cell interactions by horseradish peroxidase and endogenously generated hydrogen peroxide. ACS Chem. Biol..

[39] Liu Q., Zheng J., Sun W., Huo Y., Zhang L., Hao P. (2018). A proximity-tagging system to identify membrane protein–protein interactions. Nat. Methods.

[40] Özcelik D., Barandun J., Schmitz N., Sutter M., Guth E., Damberger F.F. (2012). Structures of Pup ligase PafA and depupylase Dop from the prokaryotic ubiquitin-like modification pathway. Nat. Commun..

[41] Guth E., Thommen M., Weber-Ban E. (2011). Mycobacterial ubiquitin-like protein ligase PafA follows a two-step reaction pathway with a phosphorylated Pup intermediate ∗. J. Biol. Chem..

[42] Yue S., Xu P., Cao Z., Zhuang M. (2022). PUP-IT2 as an alternative strategy for PUP-IT proximity labeling. Front. Mol. Biosci..

[43] Ye R., Lin Z., Liu K.-H., Sheen J., Chen S. (2023). Dynamic proximity tagging in living plant cells with pupylation-based interaction tagging. Methods Mol. Biol..

[44] Lin Z., Liu D., Xu Y., Wang M., Yu Y., Diener A.C. (2024). Pupylation-based proximity-tagging of FERONIA-interacting proteins in Arabidopsis. Mol. Cell. Proteomics.

[45] Zheng S., Noack L.C., Khammy O., De Meyer A., Khan G.A., De Winne N. (2025). Pupylation-based proximity labeling reveals regulatory factors in cellulose biosynthesis in Arabidopsis. Nat. Commun..

[46] Zheng S., Blaschek L., Pottier D., Dijkhof L.R.H., Özmen B., Lim P.K. (2025). Pupylation-based proximity labeling unravels a comprehensive protein and phosphoprotein interactome of the Arabidopsis TOR complex. Adv. Sci..

[47] Huang A., Tang Y., Shi X., Jia M., Zhu J., Yan X. (2020). Proximity labeling proteomics reveals critical regulators for inner nuclear membrane protein degradation in plants. Nat. Commun..

[48] Rugen N., Schaarschmidt F., Eirich J., Finkemeier I., Braun H.-P., Eubel H. (2021). Protein interaction patterns in *Arabidopsis thaliana* leaf mitochondria change in dependence to light. Biochim. Biophys. Acta BBA.

[49] Bao X., Jia H., Zhang X., Tian S., Zhao Y., Li X. (2024). Mapping of cytosol-facing organelle outer membrane proximity proteome by proximity-dependent biotinylation in living Arabidopsis cells. Plant J..

[50] Aravena-Calvo J., Busck-Mellor S., Laursen T. (2024). Global organization of phenylpropanoid and anthocyanin pathways revealed by proximity labeling of trans-cinnamic acid 4-hydroxylase in Petunia inflata petal protoplasts. Front. Plant Sci..

[51] Wurzinger B., Stael S., Leonardelli M., Perolo C., Melzer M., Chaturvedi P. (2022). Proximity labelling allows to study novel factors in chloroplast development^a^. bioRxiv.

[52] Kreis E., König K., Misir M., Niemeyer J., Sommer F., Schroda M. (2023). TurboID reveals the proxiomes of Chlamydomonas proteins involved in thylakoid biogenesis and stress response. Plant Physiol..

[53] De Munter S., Görnemann J., Derua R., Lesage B., Qian J., Heroes E. (2017). Split-BioID: a proximity biotinylation assay for dimerization-dependent protein interactions. FEBS Lett..

[54] Schopp I.M., Amaya Ramirez C.C., Debeljak J., Kreibich E., Skribbe M., Wild K. (2017). Split-BioID a conditional proteomics approach to monitor the composition of spatiotemporally defined protein complexes. Nat. Commun..

[55] Cho K.F., Branon T.C., Rajeev S., Svinkina T., Udeshi N.D., Thoudam T. (2020). Split-TurboID enables contact-dependent proximity labeling in cells. Proc. Natl. Acad. Sci. U. S. A..

[56] Zuo J., Niu Q.-W., Chua N.-H. (2000). An estrogen receptor-based transactivator XVE mediates highly inducible gene expression in transgenic plants. Plant J..

[57] Lee S.-Y., Lee H., Lee H.-K., Lee S.-W., Ha S.C., Kwon T. (2016). Proximity-directed labeling reveals a new rapamycin-induced heterodimer of FKBP25 and FRB in live cells. ACS Cent. Sci..

[58] Martell J.D., Yamagata M., Deerinck T.J., Phan S., Kwa C.G., Ellisman M.H. (2016). A split horseradish peroxidase for the detection of intercellular protein–protein interactions and sensitive visualization of synapses. Nat. Biotechnol..

[59] Han Y., Branon T.C., Martell J.D., Boassa D., Shechner D., Ellisman M.H. (2019). Directed evolution of split APEX2 peroxidase. ACS Chem. Biol..

[60] Kwak C., Shin S., Park J.-S., Jung M., Nhung T.T.M., Kang M.-G. (2020). Contact-ID, a tool for profiling organelle contact sites, reveals regulatory proteins of mitochondrial-associated membrane formation. Proc. Natl. Acad. Sci. U. S. A..

[61] Li J.-F., Bush J., Xiong Y., Li L., McCormack M. (2011). Large-scale protein-protein interaction analysis in Arabidopsis mesophyll protoplasts by split firefly luciferase complementation. PLOS ONE.

[62] Ren M., Venglat P., Qiu S., Feng L., Cao Y., Wang E. (2012). Target of rapamycin signaling regulates metabolism, growth, and life span in Arabidopsis[W][OA]. Plant Cell.

[63] Sormani R., Yao L., Menand B., Ennar N., Lecampion C., Meyer C. (2007). Saccharomyces cerevisiae FKBP12 binds Arabidopsis thaliana TOR and its expression in plants leads to rapamycin susceptibility. BMC Plant Biol..

[64] Winkler J., Mylle E., De Meyer A., Pavie B., Merchie J., Grones P. (2021). Visualizing protein–protein interactions in plants by rapamycin-dependent delocalization. Plant Cell.

[65] Sun Y., Li C., Deng X., Li W., Deng X., Ge W. (2024). Target protein identification in live cells and organisms with a non-diffusive proximity tagging system. eLife.

[66] Bottone S., Joliot O., Cakil Z.V., El Hajji L., Rakotoarison L.-M., Boncompain G. (2023). A fluorogenic chemically induced dimerization technology for controlling, imaging and sensing protein proximity. Nat. Methods.

[67] Benaissa H., Ounoughi K., Aujard I., Fischer E., Goïame R., Nguyen J. (2021). Engineering of a fluorescent chemogenetic reporter with tunable color for advanced live-cell imaging. Nat. Commun..

[68] Renuse S., Madugundu A.K., Jung J.H., Byeon S.K., Goldschmidt H.L., Tahir R. (2020). Signature fragment ions of biotinylated peptides. J. Am. Soc. Mass Spectrom..

[69] Festa R.A., McAllister F., Pearce M.J., Mintseris J., Burns K.E., Gygi S.P. (2010). Prokayrotic ubiquitin-like protein (Pup) proteome of Mycobacterium tuberculosis. PLOS ONE.

[70] Yu F., Haynes S.E., Nesvizhskii A.I. (2021). IonQuant enables accurate and sensitive label-free Quantification with FDR-controlled match-between-runs. Mol. Cell. Proteomics.

[71] Yu F., Teo G.C., Kong A.T., Fröhlich K., Li G.X., Demichev V. (2023). Analysis of DIA proteomics data using MSFragger-DIA and FragPipe computational platform. Nat. Commun..

[72] Liu M., Dongre A. (2021). Proper imputation of missing values in proteomics datasets for differential expression analysis. Brief. Bioinform..

[73] Goeminne L.J.E., Sticker A., Martens L., Gevaert K., Clement L. (2020). MSqRob takes the missing hurdle: uniting intensity- and count-based proteomics. Anal. Chem..

[74] Knutson S.D., Buksh B.F., Huth S.W., Morgan D.C., MacMillan D.W.C. (2024). Current advances in photocatalytic proximity labeling. Cell Chem. Biol..

